# Medical Opinion and Movement

**Published:** 1906-10-20

**Authors:** 


### Medical Opinion and Movement.

It would be difficult to over-estimate the value to humanity of the Tropical Schools of Medicine of London and Liverpool. In a surprisingly short time they have effected a revolution in the treatment of tropical diseases, have added immeasurably to the comfort of residents in the tropics, and have done much to preserve and protect human life. The Tropical School of Medicine in London is the great educational centre for tropical diseases, for the numbers of the students attending that school are about three times as numerous as those at Liverpool. The twenty-second session of the London School has an entry of forty-one students, of whom eleven are in the Colonial Service, three in the Indian Medical Service, one in the United States Army, six are missionaries, and twenty are private students, of whom two are women.

The Liverpool School of Tropical Medicine has done nearly three times as much research work as that of London. Liverpool has sent out fifteen expeditions to the tropics. Five of these were malarial, despatched to Sierra Leone, the Gold Coast, Lagos, Northern and Southern Nigeria, and Ismailia. Then there have been two yellow fever expeditions, one to Cuba and to Para in Brazil, and one to Manaos in South America; three on sanitation to Sierra Leone; three on trypanosomiasis to Gambia, French Senegal, and the Congo Free State; one to report on the sanitation and anti-malarial measures in practice at Bathurst, Konakry and Freetown, and one to the Gold Coast. The last two were sent to West Africa in appreciation of Sir William MacGregor's services to health and sanitation in that country. The publications of the Liverpool school are numerous, of great importance, and have involved a heavy drain on the resources of the school.

Mr. Jonathan Hutchinson's statements in the *Times* as to the prevalence of leprosy in the Rhone Valley are calculated to reassure intending visitors to that district. Mr. Hutchinson says emphatically that there is not the slightest cause for alarm. There are probably more lepers in London at the present time than in the whole of the Vallais Canton. In Norway the number may probably be multiplied a hundredfold, yet no one fears to reside in London or to visit Norway, and no visitor ever contracted the disease in either place. In all probability the cases in Switzerland are very few and occur only in the families of the poorest of the peasantry. The disease is known to occur all along the Mediterranean coast, and at San Remo there has long been special accommodation provided for lepers. So far as Mr. Hutchinson knows, no instance of supposed contagion has ever occurred.

A report of the Board of Health on Plague in New South Wales in 1905 has been issued by the Government. The results given in the returns are not conclusive, and several points raised have been noted for further inquiry. It appears, however, that twelve cases of plague occurred in connection with nine places of infection, and that the presence of plague-rats at each place was established. The reports are not very clear, but the conclusion to be drawn from them fairly is that the authorities may obtain due warning of a probable outbreak of plague by the continuous examination of rats. Thus the first plague rat in 1905 was identified on January 18, and the first case of plague occurred in that year on March 11. Plague was identified in rats or mice taken at twenty-eight premises, which included wharves, produce stores, private houses, two shops, and an hotel. The numbers destroyed during the whole year were 52,027 rats and 39,092 mice, a total of 91,119. Of these 28,446 were examined by the Intelligence Staff, and plague was identified in 123 rats and 18 mice.

Dr. Walsh utters some very useful and telling criticism on the extravagance of outlay which has characterised the construction of many sanatoria in England and on the Continent. He declares that to an American the elaborateness and completeness of the charity sanatoria throughout England are astonishing. They are built like the modern European hospital, with all conveniences and everything in the way of equipment that can be desired or imagined. In the case of the King's Sanatorium Dr. Walsh exclaims "that a million dollars is standing idle while the sanatorium is being built, and during the same period no patients are being treated." Contrasting this with the American system he shows that the plan there is to begin by treating patients at once in old or temporary buildings, and then to gradually extend them on modern lines by erecting new but relatively cheap structures, so that the American sanatorium gradually grows till it can accommodate all the patients requiring this kind of treatment. It is rare in America to find empty wards, but in England empty wards are very common, and the Northwood, Frimley and Pinewood Sanatoria have only half their quota of patients. This failure is due to the fact that no amount of money appears to be spared in erecting the most costly buildings for sanatorium purposes in England, but when opened they are kept half empty because there is not sufficient revenue to maintain them full. Extravagance in building is a feature of charitable sanatoria in Germany. The sanatorium at Belzig has three departments, each of which contains its own elaborate marble-tiled douche-room, magnificently equipped with every kind of douche known to modern medicine. "Everything about these rooms is beautifully nickel-plated, and one could only imagine it a room in a very luxurious and well-patronised Turkish bath." The resident medical officer told Dr. Walsh, however, that he had been there three years, but had never seen these rooms used once. Cannot the medical profession in England produce a few men of science who are also economists that the prodigal waste of money on sanatoria and hospital buildings may be stayed? Is there not one British architect who will lend his aid to achieve this pressing reform in expenditure on such buildings?

